# Development-Disrupting Chitin Synthesis Inhibitor, Novaluron, Reprogramming the Chitin Degradation Mechanism of Red Palm Weevils

**DOI:** 10.3390/molecules24234304

**Published:** 2019-11-26

**Authors:** Abid Hussain, Ahmed Mohammed AlJabr, Hassan Al-Ayedh

**Affiliations:** 1 Laboratory of Bio-Control and Molecular Biology, Department of Arid Land Agriculture, College of Agricultural and Food Sciences, King Faisal University, Hofuf 31982, Al-Ahsa, Saudi Arabia; solvia_aah@yahoo.com; 2 Research and Consulting Institute, King Faisal University, Hofuf 31982, Al-Ahsa, Saudi Arabia; 3 Ministry of Environment, Water and Agriculture, Riyadh 11442, Saudi Arabia; alayedh@kacst.edu.sa; 4 National Agriculture technology center, Life science & Environment Research Institute, King Abdulaziz City for Science & Technology, P.O. Box 6086, Riyadh 11442, Saudi Arabia; 5 RPW Consultant, United Nations, Food and Agriculture Organization (FAO), Riyadh 11442, Saudi Arabia

**Keywords:** chitin synthesis inhibitor, *chitinase*, chitin degradation, IGR, biorational insecticide, novaluron, growth retardant, *Rhynchophorus ferrugineus*, larvicide

## Abstract

Disruption in chitin regulation by using chitin synthesis inhibitor (novaluron) was investigated to gain insights into the biological activity of chitinase in red palm weevils, an invasive pest of date palms in the Middle East. Impact of novaluron against ninth instar red palm weevil larvae was examined by dose-mortality response bioassays, nutritional indices, and expression patterns of *chitinase* genes characterized in this study. Laboratory bioassays revealed dose-dependent mortality response of ninth-instar red palm weevil larvae with LD_50_ of 14.77 ppm of novaluron. Dietary growth analysis performed using different doses of novaluron (30, 25, 20, 15, 10, and 5 ppm) exhibited very high reduction in their indexes such as Efficacy of Conversion of Digested Food (82.38%) and Efficacy of Conversion of Ingested Food (74.27%), compared with control treatment. Transcriptomic analysis of red palm weevil larvae characterized numerous genes involved in chitin degradation including *chitinase*, *chitinase-3-like protein 2*, *chitinase domain-containing protein 1*, *Endochitinase-like*, *chitinase 3*, *and chitin binding peritrophin-a domain*. However, quantitative expression patterns of these genes in response to novaluron-fed larvae revealed tissue-specific time-dependent expression patterns. We recorded overexpression of all genes from mid-gut tissues. Growth retarding, chitin remodeling and larvicidal potential suggest novaluron as a promising alternate for *Rhynchophorus ferrugineus* management.

## 1. Introduction

The red palm weevil (RPW), *Rhynchophorus ferrugineus* (Olivier) (Coleoptera, Curculionidae) is an important transboundary plant pest causing huge economics losses by infesting date palm plantations in the MENA (Middle East and North Africa) region. Red palm weevils spend their early life stages within the trunk and generally take about 3–4 months to complete their life cycle [1,2]. Adult females chew palm trunk by using their long beak, and eggs are laid in these holes. After egg hatching, neonate legless larvae (grubs) feed on soft plant tissues, create feeding galleries, and start moving toward the center of palm tree. These concealed larvae (legless creamy white) grow up to 5 cm in length. RPW larvae complete their larval growth after the sixteenth instar. The larval stage lasts about 35 days in summer and 129 days in winter. Mainly larval stage is responsible for the destruction of palms. Therefore, tremendous research on the evaluation of host plant resistance [3], pheromone traps in controlling RPW [4,5], sterile insect technique [6], plant secondary metabolites [7,8,9,10], entomopathogenic nematodes [11], entomopathogenic fungi [12,13,14,15], have been done to control the invasive populations of red palm weevils worldwide. Among them, the use of different groups of insecticides as soil treatment, tree fumigation, trunk injection, wound dressing, and crown drenching of infested palms remained the focus of most of the research investigations [16,17,18,19,20].

Date palm producers in the MENA region complained RPW control failure by insecticides. Reduced susceptibility of RPW to insecticides probably because of the development of resistance [21]. The residues associated with insecticides are dangerous for the consumers and responsible for environmental pollution. Furthermore, use of synthetic insecticides is discouraged due to the deleterious effects on non-target animals. These shortcomings facilitated the policy of decision makers and research directions of researchers to sort out an innovative solution, consisting in the use of alternate target specific eco-friendly management strategies to control red palm weevils. Therefore, the development of chitin synthesis inhibitors that disrupt the chitin synthesis in the target pest species is an attractive way for chemists.

Chitin is the second most globally abundant biopolymer after cellulose found in the cuticles of arthropods, filamentous fungal cell walls, crustaceans, yeast, and algae [22,23,24]. This important cuticle structural component enables insects to fight against microbial invasion, dehydration, and physical injury. Chitin plays very important role during the growth and development of arthropods by periodic replacement of old chitin with newly synthesized chitin during molting phase [25]. Metabolism of chitin is under the strict control of chitin regulating enzymes especially chitinases. Therefore, chitin synthesis has been used as a target site for possible development of insect pests controlling products disrupting cuticle synthesis [26,27,28]. Currently, different generations of chitin synthesis inhibitors have been developed and successfully used to control pest species belongings to different orders [29]. In this regard, novaluron is a promising chitin synthesis inhibitor. Previous studies conducted against different pest species revealed the toxicity of this benzoylurea insecticide. The insecticidal potential of novaluron evaluated against *Aedes aegypti* in Brazil revealed its effectiveness against field collected organophosphate-resistant populations [30]. Recently, novaluron was evaluated against Colorado potato beetle, *Leptinotarsa decemlineata* (Say) [31]. In this study, novaluron was found to be lethal against the Colorado potato beetle by retarding the larval growth and inhibiting the chitin biosynthesis in ectodermal-derived tissues. Furthermore, novaluron exposed larvae lead to abnormal pupation and eclosion. In the past, few efforts have been made to explore the potential of chitin synthesis inhibitors against red palm weevils [32,33,34]. The scarcity of knowledge on chitin synthesis inhibitors, especially novaluron and chitin regulation in red palm weevils enabled us to conduct experimentation for the exploration of chitin metabolism at molecular level. More research on the elucidation of chitin metabolism mechanism by quantification of genes responsible for chitin degradation would greatly contribute towards effectiveness of commercial chitin synthesis inhibitor, novaluron in precisely controlling the most damaging larval instars of red palm weevil. The current study was designed for the first time by (1) evaluating the toxicity of novaluron against red palm weevil larvae; (2) larval growth and development assessment by dietary growth indices bioassays; (3) expression patterns of genes encoding chitin degradation by qRT-PCR to unfold for the first time chitin metabolism in red palm weevil larvae. The findings would contribute significantly towards target specific red palm weevils controlling products.

## 2. Results

### 2.1. Biological Activity of Different Doses of Novaluron Against Red Palm Weevil Larvae

**Figure 1:**
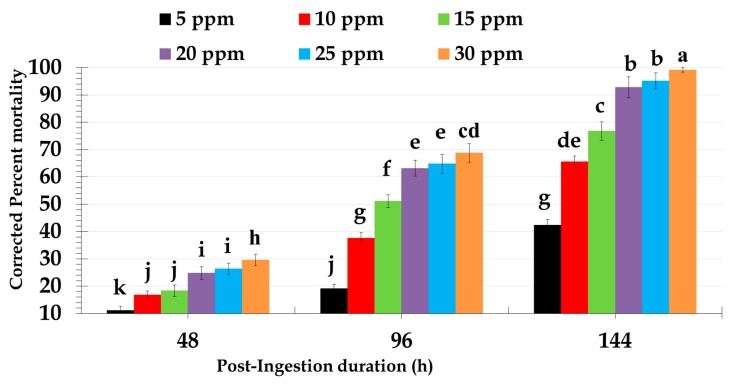
Percent corrected mortality of *Rhynchophorus ferrugineus* larvae fed on artificial diet incorporated with different doses of novaluron. Bar charts reveal means and SE calculated from five replicates. Different lower-case letter(s) above the bars indicate significant differences in the corrected percent mortality calculated at different time intervals upon feeding with different doses of novaluron. The comparison of mortalities from different treatments (doses) at different time intervals was performed by Repeated Measures ANOVA. Significant differences among the means were analyzed by Fisher’s LSD test, α *=* 0.05.

Laboratory bioassays revealed dose-dependent mortality response of ninth-instar red palm weevil larvae (Figure 1). However, significant differences in mortality of red palm weevil larvae were reported at all recorded time intervals (*F* = 146.85; *df* = 2, 60; *p* < 0.0001), using different doses of novaluron (*F* = 1032.28; *df* = 5, 60; *p* < 0.0001), and their interaction (*F* = 39.44; *df* = 5, 60; *p* < 0.0001). Artificial diet incorporated with 30 ppm of novaluron found to be the most lethal concentration imparting >95% larval mortality during experimentation (6 d). Overall, artificial diet incorporated with 30, 25, and 20 ppm of novaluron revealed >90% larval mortality. The lowest dose (5 ppm) failed to attain high mortality response and imparted 42.40% larval mortality even after 144 h post-ingestion of novaluron incorporated artificial diet (Table 1). The dietary dose of novaluron inducing 50% red palm weevil larval morality (LD_50_) was 14.77 ppm (χ ^2^ = 0.96; *df* = 4; *p* = 0.001).

### 2.2. Nutritional Indices of Red Palm Weevil Larvae in Response to Different Doses of Novaluron

Artificial diet incorporated with different doses of novaluron imparted significant variations in their dietary growth indices such as approximate digestibility (*F* = 207; *df* = 6, 28; *p* < 0.0001), efficacy of conversion of digested food (*F* = 272; *df* = 6, 28; *p* < 0.0001), and efficacy of conversion of ingested food (*F* = 196; *df* = 6, 28; *p* < 0.0001), which are vital for their growth and development of red palm weevil larvae.

The growth indices exhibited dose-dependent directly proportional pattern of ECI and ECD indexes, which showed the higher degree of reduction of these indexes with the higher dose of novaluron. Red palm weevil larvae fed on diet incorporated with 30 ppm of novaluron revealed 74.27% and 82.38% reduction of ECI and ECD indexes compared with control treatment, respectively (Table 1). The lowest dose of novaluron (5 ppm) also revealed significant reduction in ECI (9.25%), and ECD (20.54) indexes of red palm weevil larvae.

On the other hand, red palm weevil larvae fed on diet incorporated with higher doses of novaluron showed significant increase in AD (Table 1). The most potent treatment with 30 ppm and 25 ppm of novaluron in the diet that remained statistically at par with each other revealed 31.60% and 30.10% increase in AD of red palm weevil larvae compared with control treatment, respectively. The least potent dose (5 ppm) of novaluron also imparted significant increase of AD compared with control.

### 2.3. Sequence Annotations for Chitin Degradation Related Genes of Red Palm Weevils

The Expressed Sequence Tages (ESTs) of the red palm weevils were mined in the current study to compile seven clusters involved in chitin degradation (Table 2). The sequences checked against databases revealed high similarity with chitin related genes including *chitinase*, *chitinase-3-like protein 2*, *chitinase domain-containing protein 1*, *Endochitinase-like*, *chitinase* 3, and *chitin binding peritrophin-a domain* as shown in Table 2. The identified sequences with annotations mentioned here were deposited to National Center for Biotechnology Information for Accession Numbers listed in Table 2.

### 2.4. Quantitative Expression Patterns of Chitin Degradation Related Genes of Red Palm Weevils

**Figure 2:**
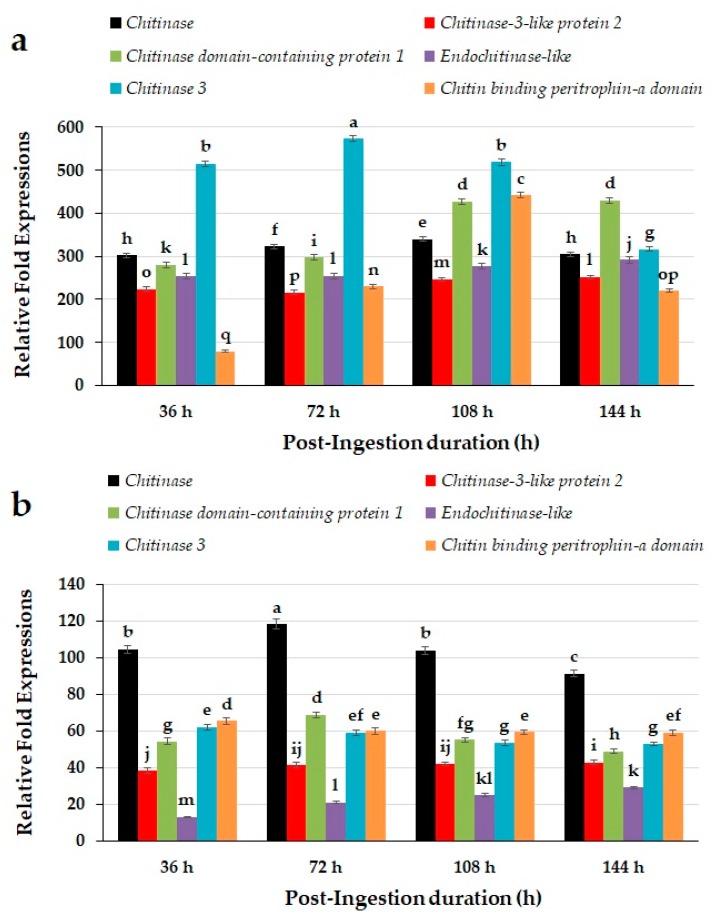
Expression patterns of red palm weevil larvae chitin degradation related genes in the (**a**) mid-gut and (**b**) carcass in response to novaluron using quantitative real-time PCR (qRT-PCR). Bar charts reveal means and SE calculated from five replicates. Different lower-case letter(s) above the bars indicate significant differences in the relative fold expressions at different time intervals upon feeding with novaluron. The comparison of relative fold expressions of different genes at different time intervals was performed by Repeated Measures ANOVA. Significant differences among the means were analyzed by Fisher’s LSD test, α *=* 0.05.

The expressions of chitin degradation related genes including *chitinase*, *chitinase-3-like protein 2*, *chitinase domain-containing protein 1*, *Endochitinase-like*, *chitinase 3*, and *chitin binding peritrophin-a domain* determined in the mid-gut of red palm weevil larvae upon ingestion with novaluron revealed time-dependent expression patterns (Figure 2a). The toxicity of novaluron revealed significant differences in the expressions of chitin degradation related genes (*F* = 1597.70; *df* = 5, 80; *p* < 0.0001), recorded at different time intervals (*F* = 54.59; *df* = 3, 80; *p* < 0.0001), and their interaction (*F* = 96.04; *df* = 15, 80; *p* < 0.0001). Among all the studied genes, *chitinase 3* greatly upregulated at 36, 72, and 108 h post-ingestion and remained statistically at the highest level. The *chitin binding peritrophin-a domain* was found to be the least expressed after 36 h of ingestion. Although, *chitinase domain-containing protein 1* and *chitin binding peritrophin-a domain* had huge difference between their expression levels, both showed similar pattern in their gene expressions. Similarly, *chitinase-3-like protein* 2 and *Endochitinase-like* also exhibited similar patterns of expression over time. On the other hand, *chitinase* remained statistically at same level except 72 and 108 h time interval, which showed slight upregulation compared with 36 and 144 h time intervals as shown in Figure 2a.

The toxicity of novaluron induced different patterns of chitin degradation related genes including *chitinase*, *chitinase-3-like protein 2*, *chitinase domain-containing protein 1*, *endochitinase-like*, *chitinase 3*, and *chitin binding peritrophin-a domain* determined in the carcass of red palm weevil larvae (Figure 2b). The carcass of red palm weevil larvae revealed significant differences in the expressions of chitin degradation related genes (*F* = 768.29; *df* = 5, 80; *p* < 0.0001), with post-ingestion time intervals (*F* = 353.82; *df* = 3, 80; *p* < 0.0001), and their interaction (*F* = 110.08; *df* = 15, 80; *p* < 0.0001). Overall, we calculated less expression of studied genes at all recorded time intervals from carcass samples of red palm weevil larvae. Among all the tested genes, *chitinase* was found to be the highly expressed gene and remained statistically at the highest level of significance compared with other chitin degradation related genes as shown in Figure 2b. On the contrary, *endochitinase-like* was found to be the least expressed gene and remained statistically at the lowest level at all recorded time intervals. However, expression patterns of *chitinase*, *chitinase domain-containing protein 1*, *chitinase 3*, and *chitin binding peritrophin-a domain* declined at the 144 h post-ingestion. On the other hand, *chitinase-3-like protein 2* and *endochitinase-like* revealed enhanced expressions over time especially at 144 h post-ingestion.

## 3. Discussion

Novaluron is an important insect growth regulator that is highly effective against immature stage of insect pest species by disrupting chitin synthesis mechanism. In the current study, novaluron was found to be toxic against red palm weevil larvae by retarding larval growth and development together with upregulation of key genes involved in chitin metabolism, which envisaged that novaluron has the potential to control *R. ferrugineus*.

This is the first study exhibiting the results of novaluron against red palm weevil larvae. However, red palm weevil larval mortalities recorded in the current study have shown great potential as pesticide. Artificial diet incorporated toxicity bioassays conducted in our study against newly molted ninth-instar red palm weevil larvae revealed that novaluron showed dose-dependent directly proportional larvicidal relationship. Our results agree with those of previous studies conducted against various pest species including *Aedes aegypti* [30], *Sparganothis sulfureana* and *Choristoneura parallela* [35], *Leptinotarsa decemlineata* [31], *Spodoptera littoralis,* and *Bemisia tabaci* [36], revealing high toxicity of novaluron. Furthermore, our findings showed 14.77 ppm of LD_50_ value for novaluron is comparable with those of commonly used red palm weevil controlling pesticides including cyprermethrin (LD_50_ value of 14.13 ppm), and ethion (LD_50_ value of 34.34 ppm) [21]. Therefore, novaluron is an effective alternative for cypermethrin and ethion replacement due to their resistance development mechanism among red palm weevil populations.

Larval growth and development that is mainly assessed on the basis of feeding indices including approximate digestibility (AD), efficacy of conversion of digested (ECD), and ingested food (ECI) are important growth parameters [9,15,37]. In the present study, disturbed larval growth and development patterns were recorded from feeding indices calculated from diet incorporated laboratory bioassays. The ninth-instar red palm weevil larvae fed on artificial diet incorporated with novaluron exhibited 31.60% increase in AD compared with control treatment larvae. Such a high increase of AD response reflected in the current study coincides with previous study on the impact of plant secondary metabolites on the feeding indices of red palm weevils [8]. Similarly, enhanced AD response reported from the grubs of red palm weevils [15], Mediterranean flour moth [38], and caterpillars of *Ocinara varians* Walker [39], suggested that nutrient-deficient exposed larvae compensate their emerging energy demands through enhancing their intrinsic abilities through AD. These findings strengthened our findings and enabled us to suggest that AD is regulated with the potency of the treatment. Highly potent treatment (30 ppm of novaluron) greatly enhanced AD activities of red palm weevils, while the lowest dose (5 ppm of novaluron) could only enhance AD to approximately 12% compared with control treatment. The less enhanced AD response is in line with previous study disclosing that non-toxic treatment failed to greatly up-regulate AD response [8].

Dietary utilization experiments on the ninth-instar red palm weevil larvae revealed tremendous reduction in ECI (74.27%) and ECD (82.38%) indexes at all tested doses of novaluron compared with the control treatment. Previous studies corroborated our findings, suggesting that the potent treatments whether plant extracts [7], conidial suspensions of entomopathogenic fungal isolates [15,37], plant secondary metabolites [8,9,10], or pesticides [21], tremendously reduced the ECD and ECI indexes. These findings enabled us to suggest that inhibition in growth indices among exposed red palm weevils arise mainly due to their depleting energy reserves. The recent findings on the exploration of sesquiterpenes clearly demonstrated that the most potent sesquiterpene (picrotoxic) disturbed the host defence mechanism. The exposed red palm weevil larvae spend most of their energy reserved on their detoxification mechanism coping with toxins. These exposed energy-deficient red lam weevils could not withstand toxins, ultimately became sluggish, growth retarded, and died [9]. Our results are also in agreement with other pest species showing disturbed growth and development patterns in case of *Helicoverpa armigera* (Hubner) against limonoids [40], *Ocinara varians* Walker against conidial suspensions [39], and *Anagasta kuehniella* against *Croton urucurana* extracts [41]. Such a tremendous reduction in ECD and ECI indexes revealed here and elsewhere in previous investigations suggest that lethality of the treatment impart detrimental impacts on the growth and development of exposed larvae resulting growth inhibition and ultimately mortality of the target host.

Chitinases are the key chitin degradation enzymes regulating the insect growth and development. Therefore, these are considered potential target site for insect pest management [42]. The transcriptomic analysis of red palm weevils unfolded the genes encoding enzymes involved in chitin metabolism. The present study for the first time revealed the characterization of the genes from red palm weevil larvae that showed homology with *chitinase, chitinase-3-like protein 2, chitinase domain-containing protein 1, endochitinase-like, chitinase 3, and chitin binding peritrophin-a domain*. The identification of *chitinase*-related multiple genes from red palm weevil larvae revealed the importance of this class of genes for pesticide development targeting chitin synthesis mechanism of different pest species. The identified genes as a result of this study would be an important genome data source for understanding biological function of *chitinase* genes especially among red palm weevils. In the past, much focus remained on the characterization of *chitinase* genes from numerous pest species due to their importance regarding the performance of specific function and their response against chitin synthesis inhibitors [42,43].

Exposure of red palm weevil larvae by novaluron incorporated diet laboratory bioassays induced different patterns of genes regulating chitin metabolism. We found that exposure of novaluron resulted in a tremendous increase in the expression of *endochitinase-like* especially from the mid-gut samples of red palm weevil larvae. Although, we observed relative decline in the expressions of *endochitinase-like* genes at the lateral stages of exposure especially at 108 h and 144 h, they remained highly expressed compared with other studied genes. Our findings are in agreement with previous study revealing the upregulation of chitin degradation genes in response to stress in the Oriental Fruit Fly, *Bactrocera dorsalis* [42]. However, *chitinase* was highly expressed in the carcass compared with other genes. Overexpression of *endochitinase-like* in the mid-gut and *chitinase* in the carcass enabled us to suggest that these genes are mainly responsible for chitin degradation. Furthermore, these genes are crucial for red palm weevil development.

In the current study, tissue specific expression patterns of chitin degradation related genes were recorded from their quantitative expression analysis. Overall, expression of all studied genes exhibited substantial upregulation from the mid-gut tissues of red lam weevil larvae. The previous studies also revealed the upregulation of *chitinase* genes and relate chitin regulation with the change in gut chitin contents [42,44]. In addition, numerous studies conducted on the insect tissue specific expression patterns of *chitinase* strengthen our findings revealing upregulation from mid-gut tissues. Similarly, *chitinase* genes identified from *Manduca sexta* also revealed the enrichment of *chitinase* in the abdomen [45]. Overexpression patterns of chitin degradation genes of red palm weevil larvae from the mid-gut tissues further suggest that mid-gut *chitinase* expressions equilibrate the mid-gut structure between synthesis and degradation. Any abrupt change due to any toxin such as novaluron used in the current study might disturb the *chitinase* expression of target pest digestive function.

Our studies clearly showed enhanced expressions of most of the target genes, which mostly downregulated at the lateral stage of exposure. On the other hand, we recorded different patterns of the expressions of the same gene from different tissues of red palm weevil larvae. The differences in their expression patterns over time and tissues are in accordance with previous studies revealing differences in their expression patterns among different tissues [46], toxicant concentration [31], and different time intervals [46]. The physiological roles of chitin degradation related genes revealed in the current study are in accordance with other studies recognizing the diverse role of *chitinase* genes in insect growth and development [42].

## 4. Materials and Methods

### 4.1. Rearing of Experimental Insects

The adults of *Rhynchophorus ferrugineus* were reared on pineapples for egg-laying. After egg hatching, third instar red palm weevil larvae were shifted on artificial diet in perforated plastic bowls at 30 ± 1 °C, 75% ± 5% relative humidity (RH) as described in detail in our previous study [37].

### 4.2. Chitin Synthesis Inhibitor

**Figure 3:**
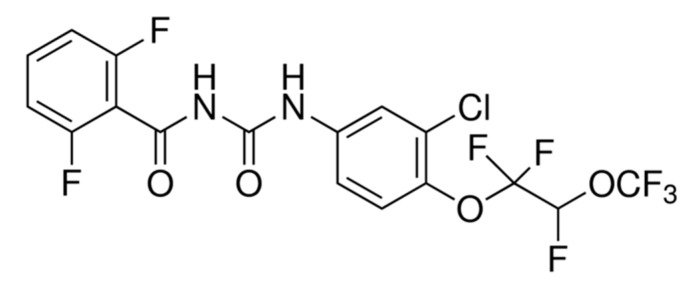
Chemical structure of novaluron.

Novaluron (1-[3-Chloro-4-(1,1,2-trifluoro-2-trifluoromethoxyethoxy)phenyl]-3-(2,6-difluorobenzoyl) urea) was purchased from Sigma-Aldrich (Cat # 32419, London., UK) as shown in Figure 3. A Stock solution of 100 mg/mL was prepared by dissolving novaluron in acetone. Different concentrations of novaluron were prepared by diluting stock solution with distilled water.

### 4.3. Biological Activity of Novaluron against Red Palm Weevil Larvae

Dose-mortality response of novaluron was evaluated against mid-aged newly molted ninth-instar red palm weevil larvae. Different doses (0, 5, 10, 15, 20, 25, and 30 ppm) of novaluron were prepared by incorporating into the artificial diet. Control treatment was prepared using respective solvent at the same concentration that was used to prepare treatment diets. A measured amount of artificial diet (2 g) was offered to each ninth-instar larva in perforated plastic bowl in an incubator at 30 ± 1 °C, 75% ± 5% relative humidity (RH). Larval mortality was recorded every 24 h for 6 d (144 h post-exposure). Each replicate was prepared by twenty-five singly fed red palm weevil larvae. Overall, five replicates each from different red palm weevil generations were prepared to compile dose-mortality response bioassay data. Observed larval mortality data were corrected from control mortality by Abbott’s formula [47]. Lethal dose to impart 50% larval mortality (LD_50_) was calculated from 96 h mortality data by Probit analysis. Angularly-transformed corrected percent mortality data were analyzed by Repeated Measure ANOVA and significant differences among means by Fisher’s LSD test [48].

### 4.4. Negative Impacts of Novaluron on Red Palm Weevil Larval Development

Novaluron at all tested doses (0, 5, 10, 15, 20, 25, and 30 ppm) was incorporated into the artificial diet to determine the impact of chitin synthesis inhibitor on the growth and development of newly molted ninth-instar red palm weevil larvae. Control treatment was prepared using respective solvent at the same concentration that was used to prepare treatment diets. Measured artificial diet was provided to the ninth-instar red palm weevil larvae in perforated plastic bowls. Each larva weight was measured before offering the diet. Each experimental unit was incubated at 30 ± 1 °C and 75% ± 5% relative humidity. Each replicate was prepared using twenty-five larvae. Five replicates were prepared likewise using five different populations. Each population was collected from different infestations. After 72 h feeding on diets, final larval weight, remaining diet and frass weight was measured. These weights were used to determine the impact of novaluron on the feeding performance of ninth-instar red palm weevil larvae by calculating efficacy of conversion of digested food [ECD = weight gained by the larva/(food ingested by the larva − dry weight of frass excreted by larvae)] and efficacy of conversion of ingested food (ECI = 100 × dry weight gained by the larva/dry weight of food consumed by larva), and approximate digestibility (AD = (food ingested − frass weight)/food ingested × 100) on dry matter basis as mentioned in previous studies [7,8,15]. Growth index data were analyzed by one way ANOVA with Fisher’s LSD test [48].

### 4.5. Analysis of Sequences Encoding Genes of Chitin Degradation Mechanism of Red Palm Weevils

The cDNA library was constructed from total RNA extracted by TRIzol (Invitrogen) reagent from the larva of red palm weevil. The first strand cDNA was synthesized by reverse transcription of mRNA using SMARTer^TM^ PCR cDNA Synthesis Kit (Clontech). The synthesized ds cDNA after purification was normalized by Trimmer-direct cDNA Normalization kit. The purified product was sent to Macrogen for Next Generation Sequencing by GS FLX platform. The sequences were assembled by Trinity v. V2.4.0. Chitin degradation related sequences were separately compiled for annotation by Blast2Go, and their annotation was confirmed by BLAST×. The sequences were deposited on NCBI database in order to make the transcriptome data publicly available.

### 4.6. Regulation of Genes Encoding Chitin Degradation Mechanism of Red Palm Weevils

Newly molted ninth instar red palm weevil larvae were fed on artificial diet incorporated with 14.77 ppm predefined dose of novaluron (LD_50_ of novaluron is 14.77 ppm) determined in the toxicity bioassays. Samples (red palm weevils) were taken after 36 h, 72 h, 108 h, and 144 h post-ingestion on artificial diet incorporated with predefined dose of novaluron. The larvae were dissected in saline to separate mid-gut and body carcass of red palm weevil larvae. Five replicates each from separate larval samples were prepared. The dissected parts were frozen in liquid nitrogen for homogenization in the presence of TRIzol reagent (Invitrogen) for total RNA extraction by RNeasy^®^ Universal Mini Kit (Qiagen). First strand cDNA of each sample was synthesized by PrimeScript First Strand cDNA Kit (Clontech) to quantify the target genes including *endochitinase*, *chitinase, chitinase-3-like protein 2, chitinase domain-containing protein 1, chitinase-like protein 1, endochitinase-like, chitinase 3,* and *chitin binding peritrophin-a domain* as shown in Table 3. The expression patterns of target genes were recorded in the CFX96 Touch^TM^ (Bio-Rad, UK) by following protocol of SYBR^®^ Premix Ex Taq. II kit (Clontech). Five replicates were prepared using five different larvae. The results of each experimental unit were compared with those of the control by relative fold expression obtained by transforming the obtained results into absolute values using 2 ^−ΔΔCt^ [49]. SAS Institute (2000) was used to determine significant differences between samples extracted from different treatments using ANOVA and Fisher’s LSD test [50].

## 5. Conclusions

In conclusion, our results indicated that novaluron is an effective insecticide, which showed larval growth inhibition and mortality in dose- and time-dependent manner, and could be incorporated into the Integrated Pest Management strategy. The identification of chitin degradation related transcriptome from red palm weevil unfolds the red palm weevil chitin degradation mechanism by exploring the expression patterns under novaluron stress. The current findings on the expression patterns of *chitinase* genes will lay the foundation, by reinforcing the idea of genome editing of *chitinase,* for the development of target specific future molecular insecticides.

## Figures and Tables

**Table 1:** Nutritional indices of *Rhynchophorus ferrugineus* larvae fed on diet incorporated with different doses of novaluron.

Novaluron Dose	Efficacy of Conversion of Digested Food (%)	Efficacy of Conversion of Ingested Food (%)	Approximate Digestibility (%)
30 ppm	6.572 ^e^	4.946 ^f^	75.363 ^a^
25 ppm	8.558 ^e^	6.302 ^e^	73.753 ^a^
20 ppm	11.240 ^d^	7.758 ^d^	69.055 ^b^
15 ppm	12.616 ^d^	8.336 ^d^	66.085 ^c^
10 ppm	17.682 ^c^	11.392 ^c^	64.437 ^c^
5 ppm	29.630 ^b^	17.440 ^b^	58.910 ^d^
Control	38.164 ^a^	19.570 ^a^	51.299 ^e^

Values within the column are the means of five replicates. Different lower-case letter(s) above the means indicate significant differences in the nutritional indices upon ingestion with different doses of novaluron. The comparison of each nutritional indices among different treatments (doses) was performed by oone way ANOVA. Significant differences among the means were analyzed by Fisher’s LSD test, α *=* 0.05.

**Table 2:** Identified Chitin degradation related genes of *Rhynchophorus ferrugineus* based on sequence similarity (*E* ≤ 10^−5^).

No	Annotation	Accession Number	Length (bp)	Expect Value
1	*Chitinase*	MK 904556	1984	1 × 10^−127^
2	*Chitinase-3-like protein 2*	MK 904557	3239	1 × 10^−172^
3	*Chitinase domain-containing protein 1*	MK 904558	4216	3 × 10^−169^
4	*Endochitinase-like*	MK 904560	1946	0.0
5	*Chitinase 3*	MK 904561	6006	0.0
6	*Chitin binding peritrophin-a domain*	MK 904562	508	8 × 10^−71^

**Table 3:** Chitin degradation related genes used to study expression patterns of the body carcass and mid-gut of *R. ferrugineus* by qRT-PCR.

Gene	Product Length	Accession Number	Forward Primer (5′-3′)	Reverse Primer (5′-3′)
*Chitinase*	73 bp	MK 904556	ACCTCTCTACGGCAGGACTT	AGCATCTGATACCGCAGCTC
*Chitinase-3-like protein 2*	85 bp	MK 904557	TCCGGTTTTCGACATGGAGG	CGTGAGGCTCTGTTCGTCAT
*Chitinase domain-containing protein 1*	88 bp	MK 904558	GTGAAGCGTTTCGCCAACAT	GCGAGGCACTAACTACGTACA
*Endochitinase-like*	99 bp	MK 904560	TCCGAACCAGTTTCCACCAG	AGTGGACGAGGGTTTTGGTC
*Chitinase 3*	94 bp	MK 904561	GCTTCTCACCACCATCCGAA	AGGTGGCTTTTCATCGTCGT
*Chitin binding peritrophin-a domain*	96 bp	MK 904562	GGGGCCCTTTTCGATGCTAA	AGACGGGGTTGACCTTGAAC
*Β-Actin*	74 bp	KM 438517	TCTATGAAGGTTACGCCCTGC	GAGGTAGTCGGTCAAGTCACG
